# Surgery

**Published:** 1894-02

**Authors:** 


### SURGERY.

EDITED BY DR. F. W. McRAE.

### ASEPTIC SURGERY IN THE COUNTRY.

Under this title Dr. J. B. Bullitt, of Arizona, has an excellent article in a late *Practitioner and News*.

We will now consider how the country surgeon can satisfy his conscience by doing aseptic surgery. Not infrequently we hear a case described, an abdominal perforating wound perhaps, to which a city surgeon was called, or which was sent to a city surgeon because the country surgeon did not have the necessary appliances to do "*antiseptic*" surgery. When the proper idea of aseptic surgery supplants the erroneous one of antiseptic, we trust the voice of this excuse will cease to be heard in the land. It is quite legitimate and proper to lack courage or skill, but no one need lack the proper means and appliances to do aseptic surgery. Coats and cuffs can be removed, sleeves can be rolled up. If cleans aprons are not handy, a clean towel can be pinned over the front. Hands and arms can be scrubbed for ten minutes in hot soap and water, particular attention being given to the nails, which have previously been cut short. The hands can now be rinsed in boiled water or alcohol, and the operator is ready. The patient can have had a bath (in set operations), or the field of operation can be thoroughly washed with soap and warm water, any hair being shaved. This can be followed by alcohol or ether. The table can be covered with a clean sheet, and the patient's body or limbs with clean towels, and table and patient are ready for operation. These sheets and towels do not need to be soaked in any antiseptic solutions. Such wet cloths cool off the patient and are unnecessary.

Von Bergmann's assistant, Schimmelbusch, in his most excellent small work on the technique of Bergmann's clinics, relates the result of the bacteriological examination of the sheets and towels which had been washed in the ordinary way by ordinary washer-women. Such sheets and towels are scrubbed and then boiled in a soda solution, afterward being ironed. This is the washerwoman's routine the world over. He found that such sheets and towels are rendered completely sterile. Of course care must be taken that no infection occurs after they leave the washerwoman's hands. In very important operations towels, etc., should be treated as the dressings are, to be hereafter described. This led to an investigation of the sterilizing properties of soda solutions. It was found that a boiling five per cent. solution of ordinary washing soda is by all odds the best sterilizing medium yet discovered. All instruments that are to be used can be boiled for *five minutes* in this soda solution, and can then be regarded as safely sterile, provided they were already ordinarily clean. If they were afflicted with concretions and the like, they must first be scrubbed with soap and water. Boiling in this soda solution also prevents rusting, and keeps instruments beautifully clean and bright. It requires no fancy piece of machinery in which to do this boiling. An ordinary fish kettle or any kind of pan or pot large enough to hold the instruments will answer perfectly. All this can be done, if necessary, in the house of the patient, or the instruments can be sterilized at home, wrapped in towels, and carried to the place they are to be used.

Dressings must be prepared at home. For them a steam sterilizer is necessary. This can be procured from any good instrument maker. The ordinary cheese cloth or butter cloth, which can be bought from dealers at five cents a yard, answers perfectly for absorbent gauze. It is cut up into desired lengths, folded, put in the sterilizer and steamed for one hour. It is then transferred to glass jars or metal boxes previously sterilized by steam or boiling and set aside until needed. The fastenings of these jars must be air-tight, so as to let in no dust. Gauze so prepared serves all the purposes of borated gauze, salicylic gauze, sublimate gauze, etc., and can be so prepared at trifling cost. If iodoform gauze is desired, it is prepared on the spot by dusting iodoform powder over this sterilized gauze. It is absolutely unnecessary to go through the troublesome process of preparing iodoform gauze with glycerine so as to render it permanently moist. Absorbent cotton, as procured from the supply man, is safe enough for ordinary use, but in very important places (abdomen, for instance) the proper thing is to put gauze, cotton, bandages (complete dressings, in other words), in the sterilizer, loosely pinned in a towel. This is steamed for an hour, allowed to dry out in the sterilizer and not opened until required for use. Such dressings can be depended upon to be absolutely sterile.

The best, safest and cheapest sponges are made from absorbent cotton and butter cloth. A bunch of cotton, larger or smaller as desired, is put on a square butter cloth cut the size of a lady's handkerchief. The four corners are tied over the cotton and the "sponge" is complete. Such balls are steamed in the sterilizer and are then ready for use. They should be used *dry;* or simple pieces of gauze cut the size of large napkins answer also very well. The cotton balls and the gauze are much better than sponges, because they are cheaper, more easily prepared and can be thrown away after being once used. The cleaning of sponges is difficult and when once infected they are always to be regarded with suspicion, and had best be thrown away.

In place of the large flat sponges, which are ordinarily considered essential in abdominal sections, Czerny, of Heidelberg, used square or rather slightly oblong pieces of heavy linen, cut about the size of a large sponge. A heavy silk thread is tied to one corner, and a pair of forceps snapped on to this when the cloth is introduced into the abdominal cavity, so there is no danger of losing it. These cloths, after being sterilized, are kept warm until ready for use. They serve to protect the intestines, which is the chief object of the broad flat sponges. Such sponges are very expensive, and the country surgeon cannot very well afford to keep a supply on hand ready for use, waiting for a case to use them on. But he can prepare the linen cloths in an hour. Czerny's outlay of dressings, etc., was very elaborate and lavish, and it was not through economy he employed these cloths, but because he found they answered the purpose better than the sponges.

This leaves only sutures, ligatures, and drainage-tubes to be considered. The small consumer will do best to buy catgut already prepared and ready for use in glass bottles. If from a good house it is fairly reliable, and saves somewhat troublesome manipulations.

Silk is to be wound on reels, glass or wood, and boiled for fifteen minutes in the aforementioned soda solution and is then preserved in tightly stoppered bottles or boxes, *dry*. If desired, it can be immersed in a five per cent. solution of carbolic acid. In important operations it is good practice to drop the silk in with the instruments and boil again immediately before use. Rubber drainage-tubes are simply prepared by boiling for *five* minutes in the soda solution, and then are permanently preserved in long glass bottles in a five per cent. carbolic solution. Now this is *all* that is required to do the most modern aseptic surgery, and is so simple and uncomplicated that it is in reach of every country surgeon who pretends to do any surgery at all. There is no necessity for deluges of water, antiseptic or otherwise. The patient's bodily warmth is thereby reduced, and the floor is converted into a pond, when it had just as well remain dry in most cases. It must be remembered there is as much danger of washing germs *into* an uninfected wound as hope of washing them out if they were there. Use just enough boiled water to remove blood stains and secure cleanliness. If fluid is required for flushing out a cavity, nothing can answer the purpose better than simple boiled water, unless indeed it be a physiological saline solution, which we are inclined to believe is, after all, the proper solution to be used in flushing out such cavities as the abdominal and pleural.

In Von Bergmann's clinic, probably the most modern and perfect in technique in Europe, the dry method of operating proposed by Landerer is used exclusively. No sponges are used. A sterilizer containing gauze cut in squares like handkerchiefs stands at hand. As the incisions are made, bleeding points are immediately caught up, the blood being taken up by the pieces of gauze, which are thrown away as fast as saturated. In this way the cut surfaces are kept absolutely dry, and when brought together at the close of the operation are in ideal condition for primary union. This dry method, when once seen, commends itself forcibly and for all time to the reason.

### THE PATHOLOGY AND TREATMENT OF APPENDICITIS.

Since laparotomy has become the *en courant* therapeutic measure as a prophylactic and radical means of cure for appendicitis, so many deaths have followed this procedure that many have searched for some constitutional remedy that might dispense with the use of the knife, in the inflammatory non-perforative type of the malady. But, before effective medication could be instituted, it was important that the underlying cause be ferreted out and that its etiology be mastered.

With this object in view, the light of modern bacteriology has been turned on, and the fluid exudate of appendicular inflammation and ulceration has been critically and systematically examined by cultures and inoculative experiments. Yet we are compelled to acknowledge that this phase of the subject remains as obscure as ever.

Hopendyl, of New York, recently examined exudate of thirty-five cases of appendicitis. In all cases he found the bacterium coli communis.

In thirty-one cases it was unassociated with any other pathogenic germ. This has been always found in other necrotic conditions of the colon; besides, in perforation of the appendix. It is said to be a germ of high pyogenic power, and when pure cultures of it are injected into the lower animal, it promptly produces local suppuration or general systemic infection.

In rare and exceptional cases of perforative appendicitis the streptococcus and staphylococcus-pyogeneus-aurens have been found. It is denied that typhoid or tubercular ulceration is ever anything more than a very rare lesion in the appendicitis. This author found concretions in but few cases of perforation, and questioned the importance of its role.

Fitz, in an analysis of three hundred cases, found concretions in but five per cent. And Ribbert, in four hundred post mortems, found fecal concretions in only thirty-eight appendices.

On the other hand, Richardson tells us, in his latest contribution on appendicitis, that he found them in all his one hundred and eighty-nine cases, at the site of perforation. He attributes the mortality in appendicitis to the bacillus coli communis, and cites Roswell Parke, who, in his essay before the late meeting of the American Surgical Society, took the same position.

In a considerable number of his cases Richardson failed to find this germ. He claims that a bacterial examination should be made of the exudate in all operations on the appendix, and that when the colon-germ is found the prognosis is unfavorable.

The observations of the New York bacteriologist, as we see, are not in accord with those of Parke; for he found the bacillus coli communis in all, indiscriminately, the mild and mortal cases, and attaches no importance whatever to it as a prognostic factor.

This germ, it should be remembered, is an inhabitant of the normal, healthy colon, and that it should be found in perforative, suppurative appendicitis in all cases is to be expected. It is known to possess migratory powers, too, for it has been found by Strauss in phagedenic bubo, and by Allen in gangrene of the scrotum. Others have discovered it in situations quite remote from the colon.

But it does not by any means appear clear how we can regard its presence in suppurative appendicitis as anything more than a fortuitous circumstance, which on no hypothesis establishes its position as an initial or causative factor in this mysterious malady.

As an operation for appendicitis is always attended with considerable danger to life, and a hernial protrusion commonly follows through the scar of the operation in successful cases, it is a question yet unsettled whether a laparotomy should ever be ventured until symptoms point to perforation.

Often the appendix cannot be found after section, and most operators are loath to make an extended search for it. Fowler, of Brooklyn, has recently reported three mortal cases, in all of which the appendix was lodged on the left of the linea-alba.—*The Times and Register.*

---

### CAMPHORATED CARBOLIC ACID.

For the last four years I have been using a preparation of camphor and carbolic acid, under the name of Carbolated Camphor, made as follows: Liquefied carbolic acid, one part by weight; gum camphor, three parts by weight. When these are put together in a bottle, the result, after a short time, is a smooth, transparent, oily liquid, almost colorless when first made, changing with age to a light pink as carbolic acid does, having the odor of camphor, a burning taste when put upon the tongue, and a specific gravity of about 0.990 compared with water. This preparation is not a chemical union but a liquefaction similar to that which takes place when chloral and camphor are mixed and there is a slight excess of camphor, which may be found in the form of a few minute crystals adhering to the bottom and sides of the bottle. It remains unchanged for an indefinite time. It mixes freely with olive oil and vaseline, and dissolves iodoform in the proportion of one to twelve, completely disguising the odor of the iodoform. It dissolves freely in alcohol, but will not mix with water or glycerine. It can be applied freely to the skin or raw surfaces without fear of cauterizing or of causing any more pain than a one-to-sixty solution of carbolic acid causes. I have used it externally, undiluted, as a general antiseptic and surgical dressing, simply saturating a piece of muslin with it and laying it over the wound or sore. The outer dressings may be taken off every day and the piece of muslin wet with the carbolated camphor without being removed, and the wound thus kept sweet and clean with very little trouble. I think I have aborted boils and cured carbuncles by injecting it around the site of the trouble as carbolic acid is sometimes used, and with less irritation than the latter causes. Mixed with carron oil in the proportion of one to twenty, I have used it with success in burns of the first and second degrees. As a domestic remedy for burns, scalds, boils, pimples, cuts, bites of insects, etc., it is excellent.

It also has great value as an internal remedy. I have used it in five cases of typhoid fever, all of which did well. In this disease I give ten to twelve drops in a capsule, every three hours, till the urine begins to show signs of carbolic acid poisoning. This generally occurs on the third day, showing that more carbolic acid can be given in this form than in the pure form. The camphor, I think, also has a good effect in this disease. When the urine begins to be affected I increase the interval between the doses to four, five or six hours, and continue giving the remedy till convalescence is well established. It must not be put into the capsule long before using or the capsule will be dissolved by it. After each dose water or diluted milk should be taken, to prevent a feeling of heat in the stomach. I have also found it very useful in many diseases of the stomach and intestine due to fermentation or the action of microbes, especially in cases of foul eructations and bad breath due to fermenting food in the stomach. It is best given in capsules, as in mixtures or emulsions the taste is disagreeable.—*Dr. D. E. English in Medical Record.*

---

### VALUE OF STRETCHING THE SPHINCTER ANI IN CHLOROFORM COLLAPSE.

In the long, sad lists of deaths, says Dr. Alexander Duke (*Lancet*, London), from chloroform in which the various means adopted for resuscitation (unfortunately ineffectual) are enumerated, I observe no mention of one of the most valuable, in my opinion, viz.: *dilatation of the sphincter ani.*

This proceeding has been, I understand, in use in America for some time past and is highly spoken of by Dr Daily, in the *New York Medical Times*, February, 1893, as effective in case of morphine poisoning.

I had lately an opportunity of putting to the test this plan of treatment in the case of a patient almost moribund after chloroform administration. The usual means having failed to obtain any response, I introduced my thumb into anus, and forcibly drew the sphincter toward coccyx. This had the immediate effect of rousing the patient sufficiently to gasp and cry out, and when repeated later on, as she showed signs of relapsing into the former condition, she so far recovered as to protest in a marked way against its repetition.

Dr. Daily's plan is to use a bivalve rectal speculum, and by its expansion to stretch the sphincter. As the speculum may not always be at hand, I think the finger (or thumb being stronger) will be found to effect the desired result.

Of course, one case does not prove much, but my observation of its immediate effect in stimulating the respiratory functions, as stated in this paper, leads me to think it a most valuable and harmless proceeding.

The sphincter ani being the last portion of the body to give up its sensibility, the converse must be equally true; hence the importance, to my mind, of adopting this plan when the patient, after an anesthetic, shows signs of collapse.

I trust that trial will be made of this American doctor's valuable suggestion, as I am convinced its importance is not known, and may be the means of saving life when the usual treatment has failed. —*Medical and Surgical Reporter.*—*St. Louis Medical Era.*

---

### INJECTIONS OF IODOFORM IN GOITRE.

Kapper (*Gaz. de Hôp.*, September 2, 1893) uses a solution containing one part of iodoform and seven parts each of ether and olive oil, which is injected into the goitre after previous disinfection of the skin. The trocar of the syringe is disinfected, and is then plunged to the depth of two or three centimeters into the tumor, and the patient is told to swallow in order to ascertain whether the canula takes part in the movements of deglutition, or whether it has not been inserted deep enough. Immediately the solution is injected, the trocar is rapidly withdrawn, and the orifice of the puncture closed by means of a piece of diachylon plaster. When the goitre is very large, he injects as much as six grammes of the solution at one sitting, in four different parts. The injections were repeated at intervals of four to six days, sometimes on several consecutive days. Local reaction was always feeble. Eight men and six women have undergone the treatment. After ten injections in the course of two months the circumference of the neck was diminished by six centimeters at least, and after another interval of two months there was a diminution of eight or ten centimeters. Besides this, the discomforts felt by the patient were sensibly attenuated. Six months after cessation of the treatment the improvement was maintained.—*Kansas City Med. Record.*

### THE RELATION OF SYPHILIS TO GENERAL PARESIS.

Dr. Frederick Peterson states that his study of this subject leads him to submit the following brief conclusions:

1. A history of syphilis is found in sixty to seventy per cent. of cases of general paralysis of the insane.
2. The fact must not be lost sight of that in thirty to forty per cent. of these cases no history of syphilis, congenital or acquired, is to be found.
3. Antecedent syphilis is seven to ten times more frequent in general paralysis than in other forms of insanity.
4. Syphilis is, therefore, to be looked upon as a frequent but not constant factor in its production.
5. But paralytic dementia is not a form of specific disease, not a late syphilitic manifestation, nor is it a form or degeneration depending upon the syphilitic poison for its origin.
6. The relationship of syphilis to general paresis lies in the facts that it is a widespread disorder in all communities, that it weakens the constitution and vitiates the blood in many whom it infects, and that the system is thus prepared in many cases for the direct operation of the final etiology factors of general paresis, viz.: alcoholism, excessive venery, hereditary and mental overstrain and excitement. —*Exchange.*

---

### THE PREMATURE ADMINISTRATION OF MERCURY IN SYPHILIS.

Mr. J. Earnest Lane (*Lancet*) protests against the administration of this drug as soon as a chancre shows a tendency to indurate. In such a sore the induration may be inflammatory in its nature, due to the application of irritating substances, and treatment by local means would be followed by healing. He believes it to be more satisfactory to await the appearances of some of the secondary sequelæ, which will render the diagnosis incontestable, and the chances for an eventual cure are improved. When mercury is administered early, the secondary manifestations are delayed, but when they do appear they are of a chronic form and by no means amenable to treatment. These patients are prone to obstinate affections of the mucous membranes and of the iris, and they are liable at a later date to develop symptoms of cerebral syphilis. It is not conclusively proven that the risk of the supervention of tertiary symptoms is in proportion to the severity and prolonged duration of the secondary stage, nor should any attempt be made to abort or shorten this stage. Mercury should be withheld until the appearance of a roseola or some other secondary lesion, and then promptly and effectually administered, preferably by inunction.—*The American Journal of the Medical Sciences.*

---

### TREATMENT OF GONORRHEA.

The author recommends the following injection, which, according to him, has given excellent results in a case of chronic gonorrhea, where sulphate of zinc, nitrate of silver and bichloride of mercury had proved inefficient. He says that he has obtained equally favorable results from this composition in the acute form of this disease:

℞. Boracic acid, ʒ iss.

Tincture of iodine, ʒ ij.

Glycerine, ℥ j.

Distilled water, q. s. ad., ℥ iv.

To be used as an injection, morning and night.—*W. S. James in International Journal of Surgery.*

